# Improved Compressive Sensing of Natural Scenes Using Localized Random Sampling

**DOI:** 10.1038/srep31976

**Published:** 2016-08-24

**Authors:** Victor J. Barranca, Gregor Kovačič, Douglas Zhou, David Cai

**Affiliations:** 1 Department of Mathematics and Statistics, Swarthmore College, 500 College Avenue, Swarthmore, PA 19081, USA; 2 Mathematical Sciences Department, Rensselaer Polytechnic Institute, 110 8th Street, Troy, NY 12180, USA; 3 School of Mathematical Sciences, MOE-LSC, and Institute of Natural Sciences, Shanghai Jiao Tong University, Shanghai 200240, China; 4 Courant Institute of Mathematical Sciences and Center for Neural Science, New York University, New York, NY 10012, USA; 5 NYUAD Institute, New York University Abu Dhabi, PO Box 129188, Abu Dhabi, UAE

## Abstract

Compressive sensing (CS) theory demonstrates that by using uniformly-random sampling, rather than uniformly-spaced sampling, higher quality image reconstructions are often achievable. Considering that the structure of sampling protocols has such a profound impact on the quality of image reconstructions, we formulate a new sampling scheme motivated by physiological receptive field structure, *localized random sampling*, which yields significantly improved CS image reconstructions. For each set of localized image measurements, our sampling method first randomly selects an image pixel and then measures its nearby pixels with probability depending on their distance from the initially selected pixel. We compare the uniformly-random and localized random sampling methods over a large space of sampling parameters, and show that, for the optimal parameter choices, higher quality image reconstructions can be consistently obtained by using localized random sampling. In addition, we argue that the localized random CS optimal parameter choice is stable with respect to diverse natural images, and scales with the number of samples used for reconstruction. We expect that the localized random sampling protocol helps to explain the evolutionarily advantageous nature of receptive field structure in visual systems and suggests several future research areas in CS theory and its application to brain imaging.

Sampling protocols have drastically changed with the discovery of *compressive sensing* (CS) data acquisition and signal recovery^1^,^2^. Prior to the development of CS theory, the Shannon-Nyquist theorem determined the majority of sampling procedures for both audio signals and images, dictating the minimum rate, the Nyquist rate, with which a signal must be uniformly sampled to guarantee successful reconstruction^3^. Since the theorem specifically addresses minimal sampling rates corresponding to uniformly-spaced measurements, signals were typically sampled at equally-spaced intervals in space or time before the discovery of CS. However, using CS-type data acquisition, it is possible to reconstruct a broad class of sparse signals, containing a small number of dominant components in some domain, by employing a sub-Nyquist sampling rate^2^. Instead of applying uniformly-spaced signal measurements, CS theory demonstrates that several types of uniformly-random sampling protocols will yield successful reconstructions with high probability^4^,^5^,^6^.

While CS signal recovery is relatively accurate for sufficiently high sampling rates, we demonstrate that, for the recovery of natural scenes, reconstruction quality can be further improved via *localized random* sampling. In this new protocol, each signal sample consists of a randomly centered local cluster of measurements, in which the probability of measuring a given pixel decreases with its distance from the cluster center. We show that the localized random sampling protocol consistently produces more accurate CS reconstructions of natural scenes than the uniformly-random sampling procedure using the same number of samples. For images containing a relatively large spread of dominant frequency components, the improvement is most pronounced, with localized random sampling yielding a higher fidelity representation of both low and moderate frequency components containing the majority of image information. Moreover, the reconstruction improvements garnered by localized random sampling also extend to images with varying size and spectrum distribution, affording improved reconstruction of a broad range of images. Likewise, we verify that the associated optimal sampling parameters are scalable with the number of samples utilized, allowing for easy adjustment depending on specific user requirements on the computational cost of data acquisition and the accuracy of the recovered signal.

Considering CS has accumulated numerous applications in diverse disciplines, including biology, astronomy, and image processing^7^,^8^,^9^,^10^,^11^, the reconstruction improvements offered by our localized random sampling may have potentially significant consequences in multiple fields. We expect that the simplicity of this new CS sampling protocol will allow for relatively easy implementation in newly engineered sampling devices, such as those used in measuring brain activity. In addition, our work addresses the important theoretical question of how novel sampling methodologies can be developed to take advantage of signal structures while still maintaining the randomness associated with CS theory, thereby improving the quality of reconstructed images.

Outside the scope of engineered devices, it is important to emphasize that we find forms of localized random sampling in natural systems. Most notably, the receptive fields of many sensory systems are much akin to this sampling protocol. In the visual system, retinal ganglion cells exhibit a center-surround-type architecture, such that the output of local groups of photoreceptors is sampled by downstream ganglion cells, stimulating ganglion cell activity in on-center locations and inhibiting activity in off-surround locations^12^,^13^. In this way, the size of the receptive field controls the spatial frequency of the information processed and the center-surround architecture allows for enhanced contrast detection. Considering the improvements in image reconstructions garnered by more biologically plausible sampling schemes, such as localized random sampling, we suggest this to be demonstration of how visual image processing may be optimized through evolution.

## Conventional Compressive Sensing Background

Due to the sparsity of natural images^14^, CS sampling, i.e., uniformly-random sampling, is typically an attractive alternative to uniformly-spaced sampling because CS renders an accurate image reconstruction using a relatively small number of samples^1^,^2^,^4^. According to the Shannon-Nyquist sampling theorem, the bandwidth of an image, which is the difference between its maximum and minimum frequencies, should determine the minimum sampling rate necessary for a successful reconstruction employing uniformly-spaced samples. The theorem demonstrates that a sampling rate greater than twice the bandwidth is in general sufficient for the reconstruction of any image^3^.

If a signal has a sparse representation in some domain, say the frequency domain, then the magnitude of many frequency components within the signal bandwidth is too small to contribute to the overall signal representation. Thus, CS theory shows that, for such sparse signals, a successful reconstruction can be achieved by using a sampling rate which is much lower than the Nyquist rate. For signals with a *k*-sparse representation in some domain, composed of a total of *k* nonzero components, CS theory shows that the sampling rate should be determined by *k* rather than the full bandwidth of the signal^1^,^2^. Since natural images are typically sparse in certain domains, a variety of coordinate transforms can be used to obtain an appropriate sparse representation viable for CS reconstruction^15^,^16^.

Since a signal is not necessarily measured in the domain in which it has a sparse representation nor is there always complete a priori knowledge of the distribution of dominant signal components, it is first necessary to identify a sparse representation of a given signal before formulating a CS reconstruction method that utilizes its sparsity. The problem of recovering a sparse signal using very few measurements takes the form of a large underdetermined system. In the language of CS theory, this is a problem of recovering an *n*-component signal, *x*, using only *m* samples, with 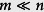, represented by an *m* × *n* sampling matrix, *A*. Each of the *m* samples is composed of various weighted measurements of the *n* signal components. Therefore, a given sample is represented by a row of the sampling matrix with each row entry containing the weight of the measurement corresponding to a specific signal component. A total of *m* such samples yields an *m*-component measured signal, *b*, which can then be used to recover the full signal. If *x* is sparse under transform, *T*, then the sparse signal representation 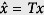 can be recovered first and then inverted to reconstruct *x*. Therefore, to recover *x* from *b*, it is necessary to solve the linear system


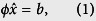


where *ϕ* = *AT*^−1^, and then compute 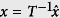.

While there are infinitely many solutions we can choose from in solving the underdetermined system (1), through adding the constraint that the solution must be sparse, CS theory aims to make this problem well-posed. By computing the solution that minimizes 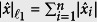 while satisfying 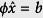, a successful reconstruction is obtainable with high probability assuming 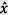 is sufficiently sparse and the sampling matrix, *A*, is appropriately chosen^1^,^6^. This is identical to solving the linear programming problem


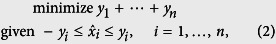


under constraint (1)^17^.

In choosing a particular sampling protocol, a key point in CS theory is that the sampling should not be uniformly-spaced. Specifically, a broad class of sampling matrices obeying the restricted isometry property (RIP) will yield a successful CS reconstruction with near certainty^4^,^5^,^6^. The sampling matrix, *A*, is said to satisfy the RIP property if 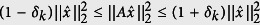 for all *k*-sparse 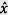 and some small *δ*_*k*_ ∈ (0, 1). For sufficiently sparse 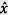 and small *δ*_*k*_, CS theory proves that the solution to (2) is an accurate representation of *x*^1^,^4^. Intuitively speaking, sampling matrices satisfying this condition preserve signal size and therefore do not distort the measured signal such that the reconstruction is inaccurate. A host of sampling matrices with independent identically distributed random variable entries, including Gaussian and Bernoulli distributed random variables, can be shown to satisfy the RIP and act as successful CS sampling protocols^1^,^4^. Intuitively, for matrices with such a random construction, a sufficient number of random measurements will lead to a relatively uncorrelated measured signal, and therefore may recover the dominant signal components with high probability. Thus, it is possible in principle to recover sparse signals since their dominant components contain the majority of information characterizing them. Measurement devices using CS-type random sampling are therefore relatively easy to design in practice and quite successful in recovering many signals^18^.

## Methods

The discovery of CS suggests that sampling protocols for natural images should shift from uniformly-spaced to random-like measurements. Are there any other sampling schemes with which we can obtain higher fidelity CS reconstructions? In the human visual system, for example, spatially nearby image features are typically processed together through neuronal receptive-fields^12^,^13^,^19^,^20^. Motivated by this structure, we consider the viability and advantage of a sampling scheme incorporating both randomness and spatial clustering, i.e., localized random sampling, in CS image reconstructions.

**Figure 1:**
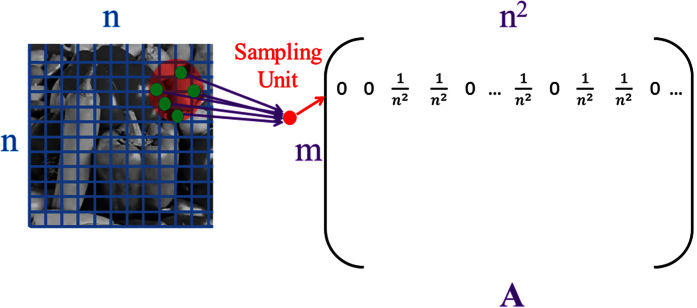
Graphical depiction of localized random sampling. Each row of the *m* × *n*^2^ sampling matrix, *A*, corresponds to localized random measurements taken by a sampling unit located at random coordinates on the [1, *n*] × [1, *n*] Cartesian grid covering the *n* × *n* pixel image. The sampling unit (located at the center of the transparent red circle) probabilistically measures nearby pixels (green dots) with sampling probability decaying with distance from its location according to Eq. (3). The sampling matrix is composed of *m* sampling units. Sampling weights are taken to be 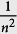 for both the localized random sampling and uniformly-random sampling protocols. Courtesy of the Signal and Image Processing Institute at the University of Southern California.

In formulating our localized random sampling methodology, we seek to recover the dominant frequency components, which capture the most relevant information regarding a natural image. To add a sufficient degree of randomness akin to conventional CS data acquisition, we first place *sampling units* on randomly chosen locations on a given image. For an *n* × *n* pixel image, choosing the location of a sampling unit is equivalent to randomly choosing coordinates on a [1, *n*] × [1, *n*] Cartesian grid, with each pair of integer coordinates corresponding to a different pixel location. Then, to obtain relevant information regarding local image features, for a given sampling unit, we measure the pixels composing the image with decreasing probability as a function of their distance from the sampling unit on the grid of coordinates partitioning the image. Thus, with respect to the sampling matrix defining the image sampling protocol, *A*, the set of measurements taken by each sampling unit corresponds to a different row of *A* with each set of spatially-clustered weighted measurements composing a row. In the case of uniformly-random sampling, however, a sampling unit instead corresponds to a row of independent identically distributed random entries in *A*, where each entry has equal probability and weight of measurement. Localized random sampling in particular prescribes that if the coordinates of the *i*^*th*^ sampling unit are (*x*_*i*_, *y*_*i*_), then the probability, *P*, to sample a pixel with coordinates (*x*_*j*_, *y*_*j*_) is given by


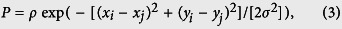


where *ρ* represents the sampling probability if (*x*_*i*_, *y*_*i*_) = (*x*_*j*_, *y*_*j*_), i.e., when the location of the sampling unit matches the location of a given pixel, and *σ* determines the radius in which the sampling unit is likely to measure image pixels. In this way, *ρ* prescribes the overall density of measurements taken by each sampling unit and *σ* specifies how closely clustered the measurements will be around the location of the sampling unit. Therefore, the location of a given sampling unit is uniformly-random, analogous to conventional CS sampling, whereas the corresponding localized pixel measurement probabilities depend on the distance of each pixel location from the sampling unit and are determined by Eq. (3). We note that, for each sampling unit and pixel location pair, *P* defines the success probability for a Bernoulli random variable determining the likelihood of pixel measurement, which is independent of all other measurements taken by the sampling unit. An illustration of the localized random sampling framework is depicted in Fig. 1. In the following sections, we demonstrate that this novel CS sampling captures both dominant low and certain moderate frequency information with a high degree of accuracy, thereby yielding significantly improved reconstructions compared with conventional uniformly-random CS sampling for a broad class of images.

Upon sampling a given *n* × *n* image using our localized random protocol with *m* sampling units, represented by *m* × *n*^2^ sampling matrix *A*, we obtain an *m*-component measurement, *b*, which we use to reconstruct the original image with *n*^2^ components. To do so, we first compute a sparse representation of the original image, e.g., the two-dimensional discrete-cosine transform. We compute the vectorization of the two-dimensional transform of the image, 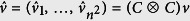, where *v* is the vectorization of the original image, ⊗ denotes the *n*^2^ × *n*^2^ Kronecker product


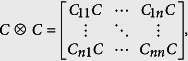


*C* is the *n* × *n*, one-dimensional discrete-cosine transform matrix with entries


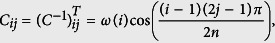


*ω*(1) = (1/*n*)^1/2^, and *ω*(*i* ≠ 1) = (2/*n*)^1/2^.

Next, we solve the CS optimization problem of recovering 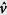 by considering the underdetermined linear system resulting from our sampling choice and sparsifying transform,


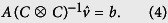


Using CS reconstruction theory, the problem of solving for 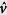 can be cast as an *L*_1_ optimization problem (2) with 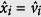 under the constraint (4). Using the Orthogonal Matching Pursuit algorithm^21^, we then recover 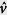. Finally, we invert the two-dimensional discrete-cosine transform and the vectorization to obtain the *n* × *n* image reconstruction. As will be shown in the subsequent section, similar results are achievable by computing the sparse image representation with the two-dimensional discrete-wavelet transform and alternative *L*_1_ optimization algorithms^22^,^23^,^24^,^25^.

**Figure 2:**
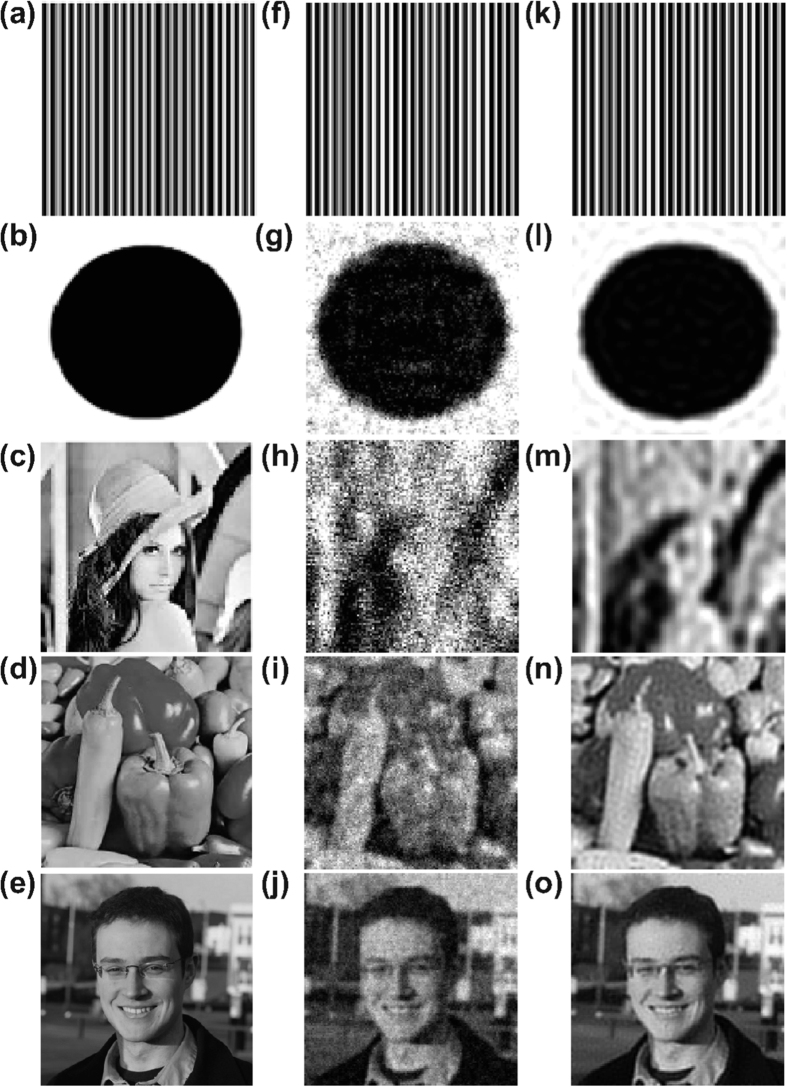
Reconstruction comparison. (**a**–**c**) 100^2^, (**d**) 250^2^, (**e**) 400^2^ pixel images. (**f**–**j**) Uniformly-random sampling CS reconstructions. (**k**–**o**) Localized random sampling CS reconstructions. For each *n*^2^ pixel image, reconstructions use *m* = *n*^2^/10 sampling units. The relative reconstruction errors via uniformly-random sampling are 1.0 × 10^−14^, 0.26, 0.40, 0.22 and 0.12 for (**f**–**j**). The relative reconstruction errors via localized random sampling are 1.3 × 10^−15^, 0.14, 0.21, 0.10, and 0.05 for (**k**–**o**). Courtesy of the Signal and Image Processing Institute at the University of Southern California.

In Fig. 2, we reconstruct three 100 × 100 pixel images of varying complexity, as well as several square images of higher resolution. To quantify the reconstruction accuracy, we compute the relative error of the reconstructed image, *p*_recon_, compared with the 100 × 100 pixel matrix representation of the original image, *p*, defined by


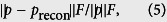


using the Frobenius matrix norm 
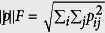
. For each image, we compare the optimal CS reconstructions using uniformly-random sampling and localized random sampling, listing the associated relative reconstruction error for each protocol in the caption of Fig. 2. Specifically, for uniformly-random sampling, we investigate the *P* parameter space and for localized random sampling, we explore the (*ρ*, *σ*) parameter space, seeking the parameter regime which yields minimal relative reconstruction error. In the case of uniformly-random sampling, each element of the sampling matrix is an independent identically distributed Bernoulli random variable with probability *P* of having a nonzero value^26^. Thus, *P* gives an indication of the density of samples taken by each sampling unit, with more measurements taken by each sampling unit for higher *P*. For both sampling matrices, we choose each nonzero entry to have magnitude 1/*n*^2^, so as to scale well with image size. Furthermore, for each 100 × 100 pixel image, we choose to use *m* = 100 sampling units for both sampling protocols, yielding a large, factor of 10, reduction in the number of samples (number of rows in the sampling matrix, *A*) compared to the number of pixels composing the original image. We similarly use a factor of 10 reduction in reconstructing the higher resolution images.

We note that for the simple striped image in Fig. 2(a), the reconstruction quality yielded by each method is quite similar and nearly perfect, i.e., very small reconstruction error, since there are relatively few dominant frequencies to recover in this case. For the more complicated disk image in Fig. 2(b), we notice a more sizeable difference in the reconstruction results corresponding to the two sampling protocols. The localized random sampling yields a reconstruction with more well-defined edges along the disk and also less noise both inside as well as outside of the disk. This noise takes the form of small areas of incorrect shade that appear in the reconstructions due to pixel (frequency) blurring, such as small white dots inside of the disk apparent in the reconstruction using uniformly-random sampling. Finally, for the most complex and natural image of Lena in Fig. 2(c), the improvement in reconstruction quality using localized random sampling is even more pronounced. While the uniformly-random sampling CS reconstruction is quite noisy and bears little resemblance to the original image, the localized random sampling yields a relatively smooth reconstruction capturing large-scale, low-frequency characteristics well. The localized random sampling reconstruction also captures some small-scale details completely missing from the uniformly-random sampling reconstruction, such as the eye of Lena and the edge of her hat. As we will show in our discussion of stability, we see even further improvements in the reconstruction quality using localized random sampling with the inclusion of higher resolution images, such as in Fig. 2(d,e), and also more sampling units. Increasing the image resolution up to 400 × 400 pixels, as in Fig. 2(e), we observe the highest quality reconstruction, with localized random sampling yielding a reconstructed image nearly indistinguishable from the original image, which holds even for detailed features and edges.

## Comparison and Analysis of Compressive Sensing Sampling Schemes

Comparing the optimal CS image reconstructions using localized random sampling and conventional uniformly-random sampling, we observed that CS using localized random sampling does indeed yield significantly more accurate image reconstructions, especially for natural images. In this section, we investigate the underlying reasons for the disparity between the two sampling methods and how they perform over a wide range of sampling parameter choices.

**Figure 3:**
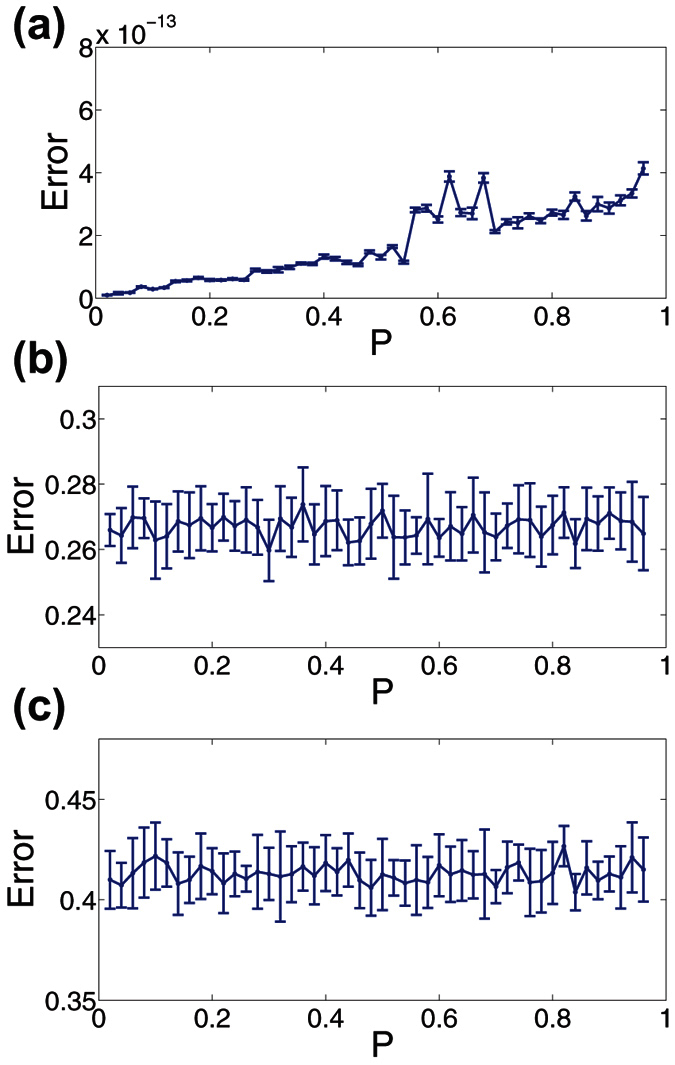
CS reconstruction relative error using uniformly-random sampling. (**a**–**c**) Reconstruction relative error dependence on measurement probability, *P*, using CS with uniformly-random sampling for the images in Fig. 2(a–c), respectively. Each reconstruction uses *m* = 1000 sampling units to recover *n*^2^ = 10000 pixel images. In each case, we do not plot the reconstruction errors near *P* = 0 or near *P* = 1 in order to accentuate error trends using more reasonable and successful sampling methodologies. For each plot, the mean relative error over an ensemble of 10 realizations of the sampling matrix for each *P* is depicted, with error bars corresponding to the standard deviation of the relative error across realizations. The parameter choices yielding minimal error are *P* = 0.02, *P* = 0.3, and *P* = 0.84 for (**a**–**c**), respectively. The mean standard deviation across realizations is 7.9 × 10^−15^, 0.0091 and 0.014 for (**a**–**c**), respectively. We note that for Fig. 3(b,c), the mean relative error is quite insensitive to changes in *P*. The minimal error corresponds to the particular *P* and corresponding sampling matrix realization for which the CS reconstruction error is lowest.

In Fig. 3, we compare the reconstruction error in recovering the images in Fig. 2(a–c) using CS with uniformly-random sampling for a wide range of sampling probabilities, *P*. Before analyzing the dependence of the reconstruction quality on *P*, it is important to remark that for all three images, as *P* closely approaches 0 or 1, the reconstruction error rapidly increases, since there are too few or too many measurements, respectively, to properly detect variations in pixel intensities of the image. Intuitively, for the very under-measured case, i.e., *P* is close to 0, only very few frequencies may be detected, failing to give sufficient information about the image. Likewise, when too many measurements are taken, i.e., *P* is close to 1, the average pixel value will primarily be detected, thereby concentrating most frequency energy near the 0-frequency. In Fig. 3, we do not plot the rapid increases in error when *P* is near 0 or 1 to focus on error trends in more reasonable parameter regimes.

In Fig. 3(a), corresponding to the stripe image, we note a slowly increasing error for larger *P* with all errors very small regardless of *P*. Since there are very few frequencies to measure it is more advantageous in this case to take fewer measurements per sampling unit, and thereby avoid averaging pixel values over the course of multiple stripes. Considering the image is simple, though, these small increases in error for larger *P* are relatively undetectable by sight.

**Figure 4:**
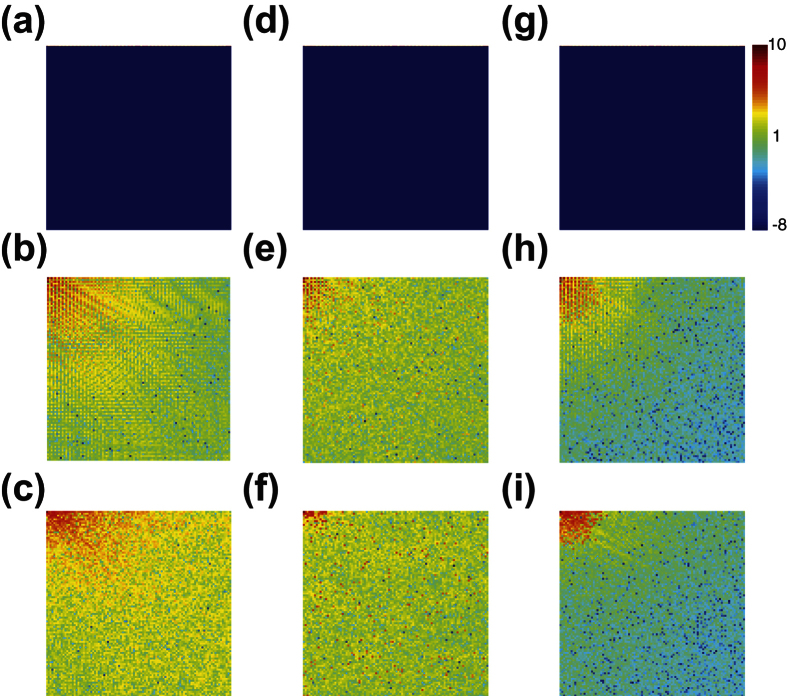
Two-dimensional discrete-cosine transform of images and their reconstructions. (**a**–**c**) Two-dimensional discrete-cosine transform of the images depicted in Fig. 2(a–c), respectively. (**d**–**f**) Two-dimensional discrete-cosine transform of the same set of images reconstructed with CS using uniformly-random sampling. (**g**–**i**) Two-dimensional discrete-cosine transform of the same set of images reconstructed with CS using localized random sampling. Each representation is computed from the natural logarithm of the absolute value of the two-dimensional discrete-cosine transform of each image, accentuating differences between lower amplitude frequencies. Note that each figure is generated using the same colorbar depicted on the upper right. The relative difference errors in low frequency amplitudes (see details in the text) corresponding to uniformly-random sampling for (**d**–**f**) are 2.8 × 10^−13^, 0.14 and 0.21, respectively. The relative difference errors in low frequency amplitudes corresponding to localized random sampling for (**g**–**i**) are 1.0 × 10^−15^, 0.05 and 0.08, respectively.

More complicated images, such as natural scenes, are typically composed of many more nonzero frequency components, resulting in a very different CS error dependence than for the simple stripes image. For comparison, we plot in Fig. 4(a–c) the frequency-domain representation of the images in Fig. 2(a–c), respectively, observing a much wider spread of dominant frequencies for the disk and Lena images. With respect to the CS reconstruction relative errors for the disk and Lena images, depicted in Fig. 3(b,c), respectively, there is largely a random spread of reconstruction errors across *P* values. For these images, since there are many more dominant frequency-components than in the case of the stripes image, taking additional measurements may capture novel frequency contributions at the cost of losing information regarding other frequency components. Hence, in this case, the density of measurements taken by each sampling unit has little effect on reconstruction quality. In fact, the maximal difference in reconstruction error is only approximately 15% across all choices of *P* and corresponding realizations of the sampling matrix. Therefore, we see that for such complicated images, the reconstruction error appears to have weak dependence on *P* as long as *P* is not near 0 or 1. Note that, for each plot in Fig. 3, the mean error is depicted over an ensemble of 10 realizations of the sampling matrix with error bars representing the standard deviation in the error. With small error bars of quite uniform size for each choice of *P*, it is clear that the CS reconstruction quality is quite stable across both realizations of sampling matrix *A* and variations in *P*.

**Figure 5:**
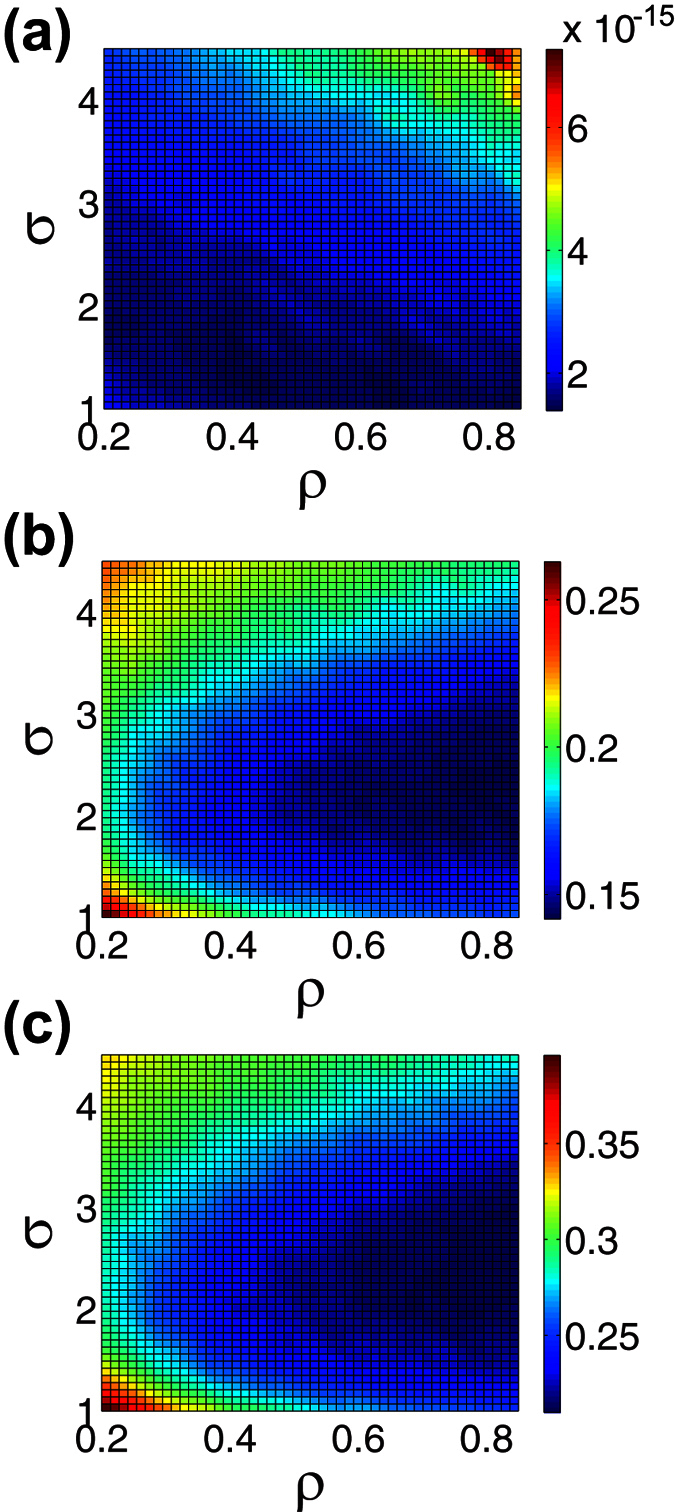
Reconstruction relative error using CS with localized random sampling. (**a**–**c**) CS reconstruction error dependence on (*ρ*, *σ*) parameter choice sets corresponding to localized random sampling of the images depicted in Fig. 2(a–c), respectively. Each reconstruction uses *m* = 1000 sampling units to recover *n*^2^ = 10000 pixel images. For each plot, the mean error over an ensemble of 10 realizations of the sampling matrix for each (*ρ*, *σ*) parameter choice set is depicted. The parameter choices yielding minimal error are (*ρ*, *σ*) = (0.88, 0.8), (*ρ*, *σ*) = (0.92, 2.2) and (*ρ*, *σ*) = (0.92, 2.2) for (**a**–**c**), respectively. The mean standard deviation across realizations in the intervals *ρ* ∈ [0.2, 0.85] and *σ* ∈ [1, 4.5] is 3.2 × 10^−16^, 0.0057 and 0.0069 for (**a**–**c**), respectively.

We similarly compute the reconstruction error dependence for the same image set using localized random sampling in Fig. 5. In each case, we vary sampling parameters defined in Eq. (3), *ρ* and *σ*, plotting the associated reconstruction error for each parameter choice. For the disk and Lena images corresponding to Fig. 5(b,c) respectively, we observe a relatively narrow area of small error, i.e., close to the minimal value, for moderately sized *σ* and high *ρ*. On the other hand, the stripes image, corresponding to Fig. 5(a), yields a relatively broad area in which the reconstruction error is close to the minimal value. It is important to note that in general the profiles of the error surfaces for images with a sufficiently high degree of frequency variation are quite similar, as we observe in comparing the example disk and Lena reconstruction errors shown in Fig. 5(b,c). Only in the case of relatively simple images, with significantly fewer dominant frequencies, do we observe relatively little variation in error across much of the (*ρ*, *σ*) space. For these simple images, as we have noted, the CS reconstruction using uniformly-random sampling can reach the same high accuracy as achieved with our localized random sampling scheme.

It is also worth noting the behavior of the localized random CS reconstruction for extreme parameter values. In all three cases, we note a rapid increase in error for extreme values of *ρ*, as in the case of sampling probability *P* for the uniformly-random sampling in Fig. 3. In the large *σ* limit with moderate *ρ*, as any pixel becomes equally likely to be sampled, we see small variation in error as the sampling procedure becomes akin to uniformly-random sampling. Likewise, for sufficiently small *σ* and *ρ*, the sampling probability at any given pixel location, including pixels located near the sampling unit, becomes vanishingly small, yielding to a similarly large increase in error as in the case of extreme *P* values.

**Figure 6:**
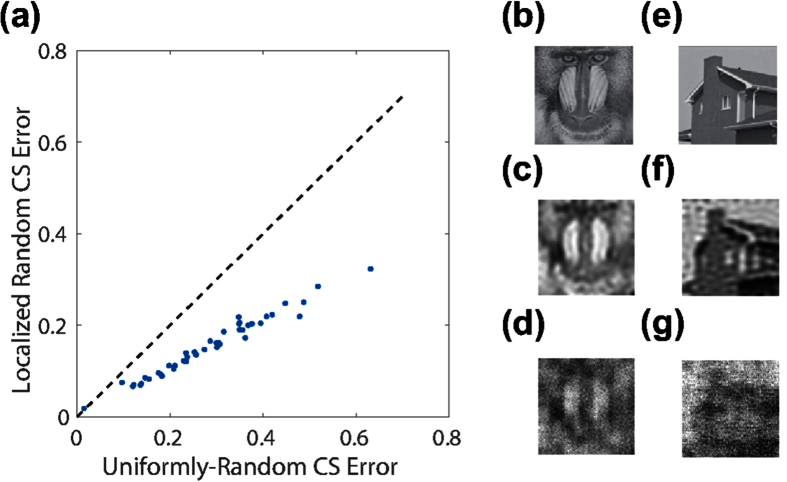
Comparison of sampling schemes over image database. (**a**) Each data point corresponds to the CS reconstruction of an image using localized random sampling (ordinate) and uniformly-random sampling (abscissa). Each localized random sampling CS reconstruction uses parameter choice (*ρ*, *σ*) = (0.92, 2.2) and each uniformly-random sampling CS reconstruction uses parameter choice *P* = 0.84. Each reconstruction uses *m* = 1000 sampling units to recover *n*^2^ = 10000 pixel images. The dashed identity line is plotted for visual comparison. There are 44 images considered, composing the University of Southern California Signal and Image Processing Institute Miscellaneous volume of images (http://sipi.usc.edu/database/database.php?volume=misc), which were processed at the 100 × 100 pixel resolution and converted to gray-scale images. (**b**,**e**) Example 100 × 100 pixel images in database. (**c**,**f**) Reconstruction of images in Fig. 6(b,e), respectively, using localized random sampling. (**d**,**g**) Reconstruction of images in Fig. 6(b,e), respectively, using uniformly-random sampling. The relative reconstruction errors via localized random sampling are 0.13 and 0.15 for reconstructions (**c**,**f**), respectively. The relative reconstruction errors via uniformly-random sampling are 0.24 and 0.29 for reconstructions (**d**,**g**), respectively. Courtesy of the Signal and Image Processing Institute at the University of Southern California.

Remarkably, for typical natural scenes, such as the Lena image, and even some less complex shapes, such as the disk, the area of small error using the localized random CS reconstruction remains approximately the same for a given number of samples, *m*. Specifically, as depicted in Fig. 5(b–c), we observe an optimal reconstruction for relatively high *ρ* ≈ 0.92 and moderate *σ* ≈ 2.2. In addition, this area of highest reconstruction accuracy is stable across realizations of the sampling matrix corresponding to each (*ρ*, *σ*) parameter choice, with very low standard deviations in each case, corroborating the robustness of this optimal parameter regime. In Fig. 6, we compare the CS reconstruction accuracy for a large database of images, primarily natural scenes, using the two sampling schemes with their respective optimal parameter choices. In nearly every case, using the same number of sampling units for each scheme, we observe that localized random sampling yields a markedly improved CS reconstruction relative to uniformly-random sampling.

Therefore, we posit that there are two fundamental reasons for the success of the localized random CS reconstruction for natural images. The first involves the structure of the spectra of these images under certain sparsifying transforms and the second is the density of samples taken by each sampling unit. Moreover, we will show in the next section that these results generalize to images of alternative resolutions and also reconstructions utilizing different numbers of sampling units.

**Figure 7:**
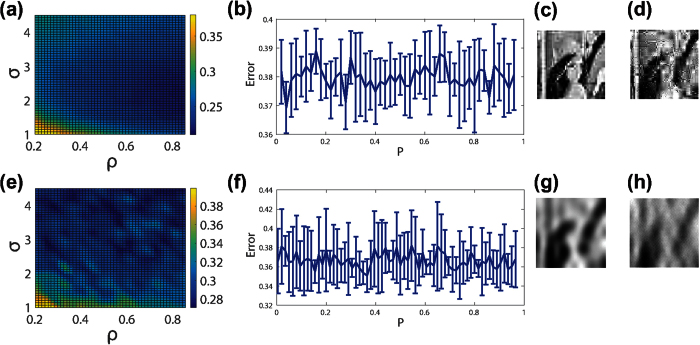
Dependence of CS reconstruction on sparsifying transformation and *L*_1_ optimization algorithm. (**a**) CS reconstruction relative error dependence on (*ρ*, *σ*) parameter choice sets corresponding to localized random sampling of Fig. 2(c) using the two-dimensional discrete-wavelet transformation. (**b**) CS reconstruction relative error dependence on measurement probability, *P*, corresponding to uniformly-random sampling for the image in Fig. 2(c) using the two-dimensional discrete-wavelet transformation. (**c**) Reconstruction of the image in Fig. 2(c) using localized random sampling and the two-dimensional discrete-wavelet transformation with the optimal sampling parameter choice. (**d**) Reconstruction of the image in Fig. 2(c) using uniformly-random sampling and the two-dimensional discrete-wavelet transformation with the optimal sampling parameter choice. (**e**) CS reconstruction relative error dependence on (*ρ*, *σ*) parameter choice sets corresponding to localized random sampling of Fig. 2(c) using the homotopy *L*_1_ optimization algorithm. (**f**) CS reconstruction relative error dependence on measurement probability, *P*, corresponding to uniformly-random sampling for the image in Fig. 2(c) using the homotopy *L*_1_ optimization algorithm. (**g**) Reconstruction of the image in Fig. 2(c) using localized random sampling and the homotopy algorithm with the optimal sampling parameter choice. (**h**) Reconstruction of the image in Fig. 2(c) using uniformly-random sampling and the homotopy algorithm with the optimal sampling parameter choice. Each reconstruction uses *m* = 1000 sampling units to recover an n^2^ = 10000 pixel image. In (**a**,**e**), the mean error over an ensemble of 10 realizations of the sampling matrix for each (*ρ*, *σ*) parameter choice set is depicted. In (**b**,**f**), the mean relative error over an ensemble of 10 realizations of the sampling matrix for each *P* is depicted, with error bars corresponding to the standard deviation of the error across realizations. The minimal reconstruction error in (**a**) is 0.21 with (*ρ*, *σ*) = (0.92, 1.9). The minimal reconstruction error in (**b**) is 0.37 with *P* = 0.04. The minimal reconstruction error in (**e**) is 0.27 with (*ρ*, *σ*) = (0.48, 4.5). The minimal reconstruction error in (**f**) is 0.35 with *P* = 0.12. The minimal error corresponds to the particular sampling parameter choice and corresponding sampling matrix realization for which the CS reconstruction error is lowest. The mean standard deviation across realizations in the intervals *ρ* ∈ [0.2, 0.85] and *σ* ∈ [1, 4.5] is 0.0089 and 0.032 for (**a**,**e**), respectively. The mean standard deviation across realizations is 0.0102 and 0.0257 in (**b**,**f**), respectively.

We emphasize that the results of this work are generalizable to other sparsifying transformations and alternate *L*_1_ optimization algorithms. For example, in Fig. 7(a,b), we consider image reconstructions using the two-dimensional *discrete-wavelet* transformation, comparing the CS reconstruction errors using localized random sampling and uniformly-random sampling, respectively^22^,^23^. As in the case of the two-dimensional discrete-cosine transformation, we observe that localized random sampling yields a higher quality optimal CS reconstruction than uniformly-random sampling. In addition, the optimal parameter choice in the localized random CS reconstruction using the two-dimensional discrete-wavelet transformation is quite close to that using the two-dimensional discrete-cosine transformation considered previously. Similarly, in Fig. 7(e,f), we consider the CS reconstruction results using the homotopy method, rather than the orthogonal matching pursuit, to solve the resultant *L*_1_ optimization problem^24^,^25^. While using a different optimization algorithm may result in alternate optimal parameter choices, we see that localized random sampling again yields improved reconstruction quality.

To investigate the reasons underlying the success of the localized random sampling, we analyze the spectra of the images in Fig. 2(a–c) and their reconstructions using each sampling method in Fig. 4. We depict in Fig. 4(a–c) the spectra of the images in Fig. 2(a–c) by taking the two-dimensional discrete-cosine transform of each image. In the case of the stripes image, we observe that the spectrum is composed of several large-amplitude frequency components in the horizontal direction, corresponding to multiples of the fundamental frequency of the stripes, whereas the remaining frequencies primarily have near-zero amplitudes. In contrast, we see that for the disk and Lena images, the spectra contain great diversity in dominant frequency-components, with varying amplitudes. As in the case of typical natural scenes, there is a high concentration of large-amplitude low-frequency components, corresponding to large-scale image characteristics, and a scatter of small-amplitude high-frequency components, corresponding to small-scale image details. It is important to note that the higher frequencies generally display lower amplitudes, and thereby typically contribute less to characterizing images.

One way to quantify the dispersion of amplitudes among the various image frequency components is to compute the image entropy, *H*, defined by


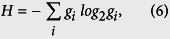


where *g*_*i*_ denotes the probability of observing the *i*^*th*^ frequency-component amplitude, computed over the set of all frequency components composing an image^27^. With their relatively wide-spread large-amplitude components, natural images should intuitively have larger image entropies than typical simpler images with only a particularly small number of dominant frequency components. In the case of the stripes image, for example, the image entropy is only 0.1418, whereas the image entropies for the disk and Lena images are 4.8996 and 5.4389, respectively. Hence, with the natural-like images having an image entropy of at least an order of magnitude greater than the simple stripes image, it is clear that such natural scenes should have significantly more diverse dominant frequencies. However, such natural images should also be distinguished from white-noise-type images, which also have high image entropy, in the sense that natural images still exhibit a concentration of image energy at lower frequencies. Images with both sufficiently high image entropies and energy concentrated in low frequency components, therefore, appear to be good candidates for the large improvements in reconstruction quality yielded via localized random sampling.

In Fig. 4(d–f), we plot the two-dimensional discrete-cosine transform of the same respective images reconstructed using uniformly-random sampling CS reconstructions. We note that, for the simple stripes image, the spectrum in Fig. 4(d) is nearly identical to the spectrum of the original image. In addition, as displayed in Fig. 2(f), the corresponding image reconstruction in the space domain is also highly accurate. For the other images, we observe larger differences in the transforms of the reconstructions, plotted in Fig. 4(e,f), relative to the original image transforms. While the relative amplitudes of the low and high frequency components in Fig. 4(e,f) are similar with respect to the original images, the clear patterning in the amplitudes of the higher frequency components is lost in the uniformly-random sampling CS reconstructions. In addition, especially in Fig. 4(f), the distribution of several large-amplitude low-frequency components appears slightly distorted, contributing to errors in resolving certain large-scale image features. These spectral differences correspond to a lack of accurate higher-order image information, which may cause the graininess observed in the images reconstructed in Fig. 2(g,h).

For comparison we plot in Fig. 4(g–i) the corresponding two-dimensional discrete-cosine transform of the CS reconstructions using localized random sampling. Again, we note nearly perfect recovery of the spectrum for the stripes image in Fig. 4(g). For the transforms of the reconstructed disk and Lena images displayed in Fig. 4(h,i) respectively, we note a patterning of the dominant frequency-component amplitudes relatively similar to the corresponding original images yet distinct from the transforms of the reconstructions using uniformly-random sampling. Comparing the spectra of the Lena image, for example, the frequency-components of the CS reconstruction using uniformly-random sampling depicted in Fig. 4(f) are less dominant in the vertical direction than the same components corresponding to both the original image and localized random sampling CS reconstruction in Fig. 4(c,i), respectively. Likewise, in the case of the disk image, the contribution of several low-frequency components dominant in the original image and localized random sampling CS reconstruction are instead diminished in the uniformly-random sampling CS reconstruction. We also note that the higher-frequency components have primarily near-zero amplitudes in the localized random sampling CS reconstructions, typically yielding sparse representations in the two-dimensional discrete-cosine transform domain.

Overall, we observe especially good agreement between the localized random sampling CS reconstructions and original images for low and moderately-high frequency components. We quantify this agreement in the spectra by using the Frobenius norm relative error in frequency amplitudes, as analogously defined in Eq. (5) in the case of image pixel matrices. We compute this relative error for the frequency-amplitude matrix representations of the images and their reconstructions. In making the comparison, to better quantify agreement in the relative distribution of the frequency amplitudes, we first normalize the frequency amplitudes of the compared images by their respective maximum frequency-amplitudes. Computing this relative spectral difference for the 20 × 20 submatrix of amplitudes corresponding to combinations of the 20 smallest positive *x* and *y* frequency components, we observe closer spectral agreement between the original images and their CS reconstructions for both the disk and Lena images by using localized random sampling. The exact values of these relative differences, using uniformly-random and localized random sampling, can be found in the caption of Fig. 4. Since the highest frequency components make little contribution to the overall image features, improved agreement in the lower frequencies via localized random sampling typically results in much greater improvement of the image reconstruction than a comparable improved agreement in high-frequency contributions.

Considering each sampling unit will generally measure groups of spatially nearby pixels via localized random sampling, the approximate average pixel intensity in the region of each sampling unit and the corresponding frequency information characterizing the variation among those pixels should be well captured. Also, since the sampling units are each randomly placed on the image, the groups of spatially nearby pixels measured by the sampling units are expected to be uniformly spread across the image. Additional frequency information may thus be acquired through the difference in intensities between distinct clusters of measured pixels. With respect to the highest frequency component contributions, we observe much lower amplitudes in the spectra of the localized random sampling CS reconstructions relative to the corresponding uniformly-random sampling CS reconstructed image spectra in Fig. 4. By utilizing localized random sampling, we expect that a lack of intersections between clusters of measured pixels may cause high-frequency contributions to be missed as the price for better resolution of lower frequencies, which we will later discuss further with respect to the density of samples taken by the sampling units.

Overall, we see that localized random sampling yields a better reconstruction of low and moderately-high frequency components, which contribute most to the overall image features and reconstruction accuracy. Similarly, in pixel space, capturing these dominant frequencies well corresponds to improved resolution of small-scale features and abrupt transitions in pixel intensity, which are often missed through uniformly-random sampling with the same number of sampling units, as evidenced in Fig. 2.

In addressing the question of what determines the success of localized random sampling, we now investigate how dense the measurements of each sampling unit should be for a natural image. While sampling density did not appear to impact the success of CS reconstructions using uniformly-random sampling for moderate values of *P*, as shown in Fig. 3, we observe a clear dependence on sampling probability parameter, *ρ*, in the localized random sampling scheme, as shown in Fig. 5. To quantify the number of measurements taken by each sampling unit, we measure the sparsity of each sampling matrix. We define the sparsity of a matrix to be the percentage of zero-component entries that are contained in the matrix. Thus, sampling units taking very few measurements will have a sparsity near 1.

**Figure 8:**
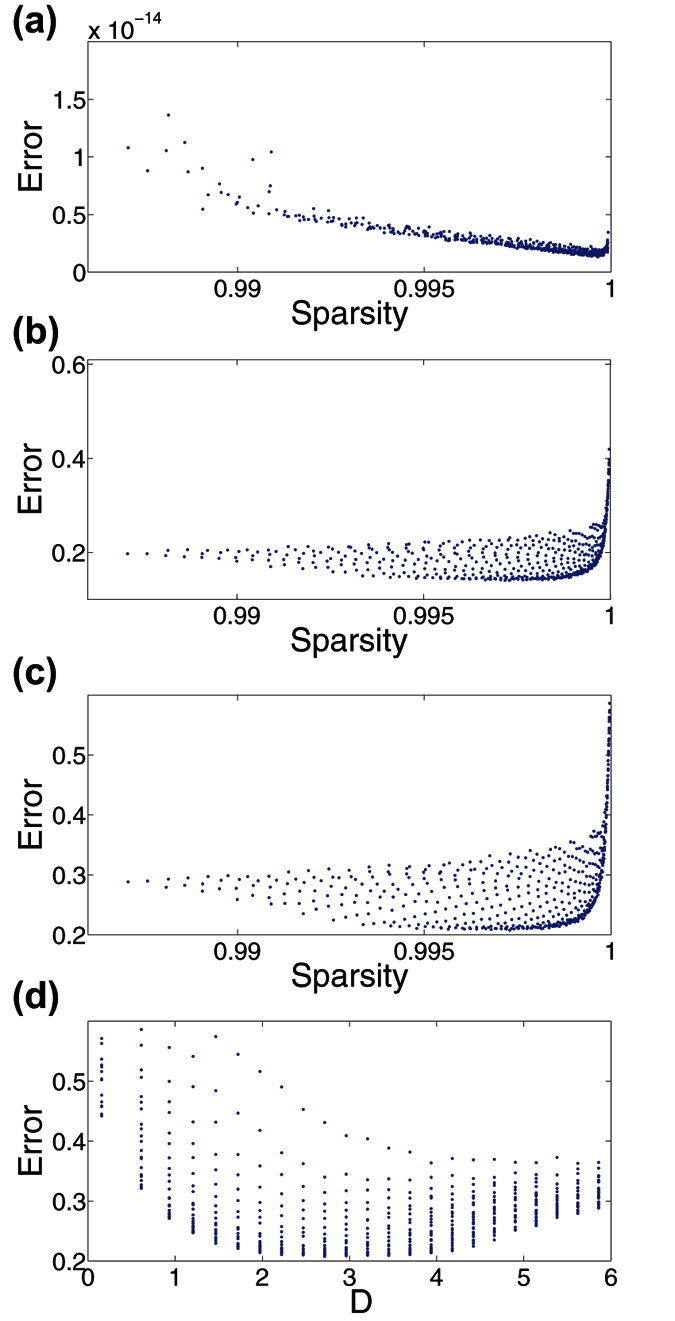
Impact of measurement sparsity on reconstruction relative error. (**a**–**c**) Dependence of the CS reconstruction relative error on sampling matrix sparsity using localized random sampling for the images depicted in Fig. 2(a–c), respectively. Plots (**a**–**c**) depict reconstruction errors corresponding to each of the (*ρ*, *σ*) parameter choices and resultant sampling matrix sparsities used in Fig. 5(a–c), respectively. For each plot, the mean error and mean sparsity over an ensemble of 10 realizations of the sampling matrix for each (*ρ*, *σ*) parameter choice set is depicted as a point. The sparsity values corresponding to the minimal reconstruction errors for Fig. 2(a–c) are 0.9996, 0.9973, and 0.9973, respectively. (**d**) CS reconstruction relative error as a function of the expected distance between sampling units and sampled pixels, *D*, defined by Eq. (7), for each (*ρ*, *σ*) parameter choice using localized random sampling in reconstructing Fig. 2(c). The mean relative error over an ensemble of 10 realizations of the sampling matrix for each (*ρ*, *σ*) parameter choice set is plotted as a point for each corresponding value of *D*. The expected distance that corresponds to the minimal reconstruction error plotted in (**d**) is *D* = 2.17. We note that multiple (*ρ*, *σ*) parameter choices may yield the same sparsity or expected distance, but generate quite different reconstruction errors.

In Fig. 8(a–c), we plot the dependence of the reconstruction relative error on sampling matrix sparsity for each of the (*ρ*, *σ*) parameter choices used in Fig. 5. For each image, the minimal errors are clustered near a sparsity of 0.999, with increasingly large errors in both the low and high sparsity limits. In the extremely low and high sparsity regimes, low quality reconstructions are yielded for similar reasons as previously summarized in the discussion of extreme parameter choices for localized random sampling. However, what is significant is that the optimal sparsity values approximately correspond to 1 − 1/*m*, where *m* is the number of utilized sampling units. Since the total number of elements in the *m* × *n*^2^ sampling matrix is *mn*^2^, if the fraction of nonzero elements is 1/*m*, then the expected number of total pixels measured is *mn*^2^(1/*m*) = *n*^2^, which exactly equals the total number of pixels in the image. In this particular case, each pixel is sampled approximately once. Hence, there is statistically little over-sampling across sampling units, such that the contributions of measured pixels (frequencies) blur, and also little under-sampling, such that the contribution of specific pixels (frequencies) are missing. For example, in the case of the previous reconstructions with *m* = 1000 sampling units used to reconstruct *n*^2^ = 10000 pixel images, the optimal sparsity is near 1 − 1/*m* = 0.999.

We note that over-sampling in this sense is distinct from adding rows (sampling units) to the sampling matrix, which would be expected to improve the image reconstruction. Here, over-sampling refers to the rows of the sampling matrix *A* having too many non-zero entries, and thus each sampling unit takes too many measurements, such that there tends to be redundancy in the spectral information yielded by each sampling unit. Likewise, if too few measurements are taken by each sampling unit, certain pixels (frequencies) may never be measured and thus less information will be available for reconstruction. In contrast, given the optimal sparsity of the sampling matrix *A*, since each pixel is expected to be sampled only once, it is most likely for each sampling unit to collect sufficient but not redundant image information.

For more dense measurements, i.e., large *σ* for fixed *ρ*, in which the localized clusters of pixels corresponding to each sampling unit tend to have more overlap, the spectra of the reconstructions using localized random sampling CS become more similar to the spectra of reconstructions using uniformly-random sampling. In the extreme case that 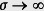, localized random sampling reduces to uniformly-random sampling, and thus the spectra of the reconstructions become the same as in the uniformly-random sampling case. We suspect that by using localized random sampling with more dense measurements, the overlaps between clusters of measured pixels may resolve a few higher frequencies at the price of missing low-frequency component contributions, which are typically more significant. In the case of the optimal sparsity, however, the expected lack of localized pixel cluster intersections likely corresponds to less resolution of higher frequencies, but improved resolution of lower frequencies through distinct local measurements. Since lower frequency components contain the most vital image information, improving their resolution produces higher quality reconstructions corresponding to the minima in Fig. 5(b,c). We demonstrate this by determining the expected distance between a given sampling unit and a sampled pixel for each localized random sampling CS reconstruction parameter choice. When this distance is greater, it is clearly more likely that the clusters of measured pixels corresponding to each sampling unit will intersect. Averaging across all sampling unit locations and possible sampled pixels, we compute for each (*ρ*, *σ*) parameter choice the expected distance between the sampling units and sampled pixels, *D*, given by


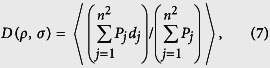


where the probability of connection, *P*, is determined by Eq. (3), index *j* corresponds to the *j*^*th*^ possible pixel location on the image lattice, *d* gives the Euclidean distance between a sampling unit and pixel to be measured, and 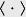 corresponds to the expectation over all possible sampling unit locations on the image lattice. In Fig. 8(d), we plot the CS reconstruction errors displayed in Fig. 5(c) as a function of *D* for each (*ρ*, *σ*) parameter choice. We observe a clear minimal error for intermediate *D*, giving evidence for an optimal cluster size, corresponding to the optimal sampling matrix sparsity at which each pixel is expected to be sampled approximately once.

In summary, both the distribution of dominant frequencies in image spectra and the sparsity of measurements taken by sampling units play a fundamental role in the success of image reconstructions using localized random sampling. For natural images, in which there is sufficient variation in dominant frequencies, localized random sampling resolves low and moderately-high frequency information especially well, characterizing the majority of image features. Likewise, when the sparsity of the sampling matrix is approximately 1 − 1/*m*, there is little overlap between clusters of sampled pixels, allowing most sampling resources to be used towards resolving lower frequency information while still containing some high-frequency information from measurements within clusters of measured pixels.

## Stability of Localized Random Sampling

The analysis thus far has focused on pixel images of the same 100 × 100 resolution reconstructed using a constant number of *m* = 1000 total samples. We conclude by studying how well our results generalize to images of other resolutions and also CS reconstructions using different numbers of sampling units. In particular, we identify and explain differences in reconstruction quality and optimal parameter choices that may arise in each of these alternative scenarios.

**Figure 9:**
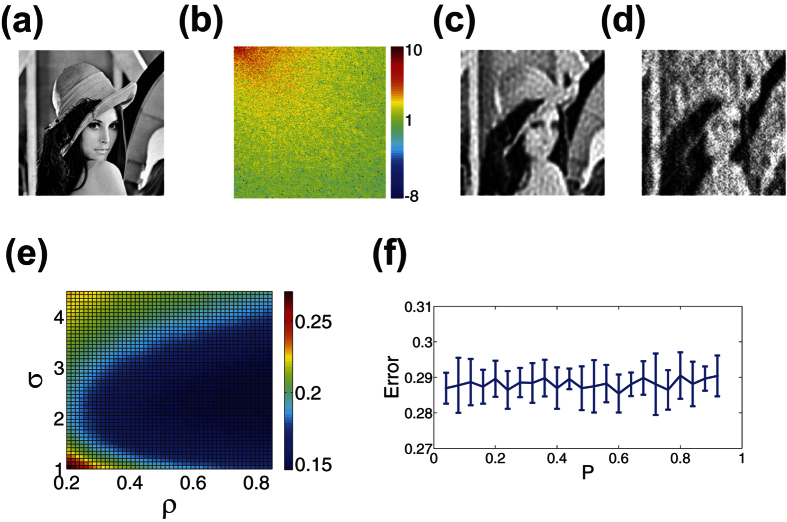
CS reconstructions for larger images. (**a**) 200 × 200 pixel image of Lena. (**b**) Two-dimensional discrete-cosine transform of image (**a**). This representation is computed from the natural logarithm of the absolute value of the two-dimensional discrete-cosine transform of image (**a**). (**c**) Optimal CS reconstruction of image (**a**) using localized random sampling. (**d**) Optimal CS reconstruction of image (**a**) using uniformly-random sampling and the same number of sampling units as in (**c**). (**e**) CS reconstruction relative error dependence on (*ρ*, *σ*) parameter choice sets corresponding to localized random sampling of image (**a**). (**f**) CS reconstruction relative error dependence on measurement probability, *P*, using uniformly-random sampling of image (**a**). Each reconstruction uses 4000 sampling units to recover a 40000 pixel image. In reconstruction (**c**), a minimal reconstruction relative error of 0.14 is achieved using the parameter choice (*ρ*, *σ*) = (0.96, 2.5). In reconstruction (**d**), a minimal reconstruction relative error of 0.28 is achieved using *P* = 0.6. In (**e**), the mean relative error over an ensemble of 10 realizations of the sampling matrix for each (*ρ*, *σ*) parameter choice set is depicted. The mean standard deviation across realizations in the intervals *ρ* ∈ [0.2, 0.85] and *σ* ∈ [1, 4.5] is 0.0026. In (**f**), the mean relative error over an ensemble of 10 realizations of the sampling matrix for each *P* is depicted, with error bars corresponding to the standard deviation of the error across realizations. The mean standard deviation across realizations in (**f**) is 0.0055.

To address the issue of image resolution, we consider a 200 × 200 pixel image of Lena in Fig. 9(a) analogous to the 100 × 100 pixel version depicted in Fig. 2(c). In Fig. 9(c,d), we reconstruct this larger image with *m* = 4000 sampling units using localized random sampling and uniformly-random sampling, respectively. Note that we use the same factor of 10 fewer sampling units than total pixels we seek to recover, as in the previous analysis.

For both sampling protocols, we observe a significant improvement in reconstruction quality relative to the corresponding 100 × 100 pixel image reconstruction. The reason for this improvement can be explained by comparing the spectra of the different-sized Lena images. First, it is clear that the two-dimensional discrete-cosine transform of this larger Lena image, depicted in Fig. 9(b), is very similar in overall structure to the transform corresponding to the smaller Lena image, depicted in Fig. 4(c). We observe in Fig. 9(b) that while some higher-frequency components are introduced in the higher-resolution image, the distribution of amplitudes in the dominant low-frequency components is nearly indistinguishable from that of the lower-resolution Lena image. In particular, the relative frequency amplitude difference between the low frequency components for the two images, as defined previously, is only 0.002. For comparison, we note that the frequency amplitude difference in the case of two completely different images of the same resolution is several orders of magnitude larger. For example, the frequency amplitude difference between the Lena and disk images in Fig. 2(b,c) has a value of 1.01. As shown in Fig. 9(b), the newly introduced high-frequency components have very small amplitudes and thus have relatively little impact on the overall image features compared to the lower frequency components. Since maintaining the same ratio of sampling units to recovered pixels for higher resolution images requires increasing the total number of sampling units utilized, these additional sampling units may greatly increase the accuracy of image reconstructions, especially if the new samples resolve the dominant low-frequency-component contributions well. We see from Fig. 9(c) that the improved resolution of low-frequency contributions utilizing these additional localized random samples does indeed greatly improve the quality of the recovered image.

Comparing the optimal reconstructions using localized random and uniformly-random sampling in Fig. 9(c,d), respectively, we still observe a much higher degree of accuracy is achieved via localized random sampling for the 200 × 200 pixel image. Localized random sampling allows for the resolution of even smaller-scale details than in the case of the corresponding smaller Lena image reconstructed in Fig. 2(m), capturing features as fine as the nose and mouth of Lena, which were mostly missing in the smaller image recovery. We compare the reconstruction errors over a range of sampling parameter choices in Fig. 9(e,f) using localized random and uniformly-random sampling, respectively. In the case of uniformly-random sampling, the distribution of errors is again quite unaffected by variations in sampling probability *P*. Using instead localized random sampling, the smallest reconstruction errors are yielded by utilizing approximately the same sampling parameters as in the case of the smaller 100 × 100 pixel image. Specifically, (*ρ*, *σ*) = (0.96, 2.5) and (*ρ*, *σ*) = (0.92, 2.2) yield minimal reconstruction relative errors for the 200 × 200 and 100 × 100 pixel images, respectively, with nearby parameter choices producing quite high reconstruction quality for natural images of either resolution. Thus, for natural scenes varying in resolution, the characteristics of the localized random sampling protocol are closely related.

**Figure 10:**
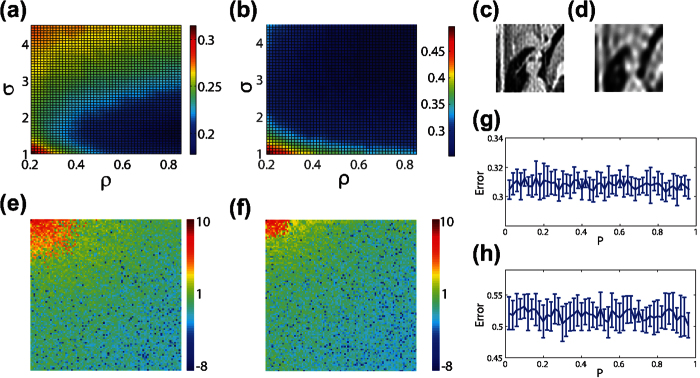
Dependence of CS reconstruction quality on number of sampling units. (**a**,**b**) CS reconstruction error dependence on (*ρ*, *σ*) parameter choice sets corresponding to localized random sampling of the 10000 pixel image in Fig. 2(c) using *m* = 2000 and *m* = 500 sampling units, respectively. (**c**) Optimal CS reconstruction using localized random sampling with *m* = 2000 sampling units. (**d**) Optimal CS reconstruction using localized random sampling with *m* = 500 sampling units. (**e**,**f**) Two-dimensional discrete-cosine transform of the reconstructions in (**c**,**d**), respectively. (**g**,**h**) CS reconstruction relative error dependence on measurement probability, *P*, using uniformly-random sampling for the image in Fig. 2(c) with *m* = 2000 and *m* = 500 sampling units, respectively. Each transform is computed from the natural logarithm of the absolute value of the two-dimensional discrete-cosine transform of each image. The relative reconstruction errors corresponding to (**c**,**d**) are 0.17 and 0.25, respectively. The corresponding optimal parameter choices are (*ρ*, *σ*) = (0.96, 1.75) and (*ρ*, *σ*) = (0.92, 4.5) respectively. We note that the minimal reconstruction errors using uniformly-random sampling corresponding to *m* = 2000 and *m* = 500 sampling units are 0.30 using *P* = 0.84 and 0.50 using *P* = 0.96, respectively. In (**a**,**b**), the mean relative error over an ensemble of 10 realizations of the sampling matrix for each (*ρ*, *σ*) parameter choice set is depicted. The mean standard deviation across realizations in the intervals *ρ* ∈ [0.2, 0.85] and *σ* ∈ [1, 4.5] is 0.0049 and 0.0086 for (**a**,**b**), respectively. In (**g**,**h**), the mean relative error over an ensemble of 10 realizations of the sampling matrix for each *P* is depicted, with error bars corresponding to the standard deviation of the error across realizations. The mean standard deviation across realizations is 0.0076 and 0.023 in (**g**,**h**), respectively.

With respect to the number of samples (number of rows in the sampling matrix *A*) utilized, we consider the cases in which *m* = 2000 and *m* = 500 sampling units are used, doubling and halving, respectively, the number of sampling units employed in the previous section. In Fig. 10(a,b), we plot the CS reconstruction error using localized random sampling of the small 100 × 100 pixel Lena image depicted in Fig. 2(c) over a wide range of (*ρ*, *σ*) parameter choices. We again note a distinct region of minimal reconstruction error, but the minimum is slightly shifted with varying choices of *m*. We hypothesize that the reason for this small shift is because with larger numbers of samples, there is the opportunity to identify, without loss, higher, less dominant, frequency components. Thus, as the number of sampling units increases, the optimal radius in which pixels should be sampled, corresponding to the size of parameter *σ*, should decrease to avoid overlap between distinct clusters of measured pixels. While the limit of this case will be the same as the uniformly-random sampling with sufficient number of sampling units, using such a large number of samples diminishes the sampling efficiency garnered by CS theory.

To demonstrate the relationship between the number of sampling units and recovered image frequencies, we plot in Fig. 10(c,d) the optimal image reconstructions and their associated two-dimensional discrete-cosine transforms in Fig. 10(e,f), using localized random sampling with *m* = 2000 and *m* = 500 sampling units, respectively. It is clear that the transform corresponding to the larger number of sampling units contains a broader distribution of large-amplitude components, including higher frequencies. Moreover, more sampling units also yields more accurate resolution of low-frequency amplitudes. Thus, in pixel space, there is a marked improvement in reconstruction quality by using more sampling units. In practice, depending on the available computing resources and desired reconstruction quality, the number of utilized sampling units can be adjusted accordingly. For comparison, we plot in Fig. 10(g,h) the CS reconstruction error using uniformly-random sampling over the sampling probability parameter space corresponding to the same respective numbers of sampling units. As in the previous cases, the optimal CS reconstruction quality is greatly improved by utilizing localized random sampling.

It is important to note that for an appropriately chosen *σ*, a high *ρ* of approximately 0.9 will typically yield an accurate reconstruction using image sizes for which available computing resources allow recovery. We expect that for increasingly large numbers of sampling units utilized, *σ* should be appropriately adjusted so as to maintain the optimal measurement rate such that each pixel is approximately sampled once. While we see that the optimal *σ* decreases with the number of sampling units used, further research is necessary to quantitatively describe this trend in more general cases. However, if too many sampling units are utilized, it is clear that the benefits of reduced sampling rates garnered by CS are diminished, making such a scenario less useful to consider. Likewise, if too few sampling units are used, then the expected cluster size will need to be quite large, giving reconstruction results similar to the uniformly-random sampling CS reconstruction. Hence, as demonstrated in Fig. 10(b), when particularly few sampling units are used, larger *σ* values typically yield improved reconstructions. Overall, utilizing a particularly small number of samples of natural image pixels, the localized random sampling protocol demonstrates a relatively stable dependence on parameter choices and reconstruction algorithms, and therefore is quite robust in suiting diverse applications.

## Discussion

In this work, we have formulated and analyzed a new sampling methodology, motivated by the structure of sensory systems, which is viable for improved CS image reconstructions. Using our localized random sampling, consisting of sets of randomly chosen local groups of sampled pixels, we recovered a variety of images using compressive sensing techniques. We demonstrate that, especially for natural scenes, this new sampling protocol yields remarkably higher quality image reconstructions than more conventional uniformly-random pixel sampling. Using spectral analysis, we conclude that sampling localization better resolves lower-frequency components, which contain more information regarding image features, while retaining some higher frequency information. Moreover, we also showed that the optimal parameter choices corresponding to this new sampling protocol are stable with respect to variations in natural image sizes and may be slightly adjusted according to the number of samples utilized. Relatively easy to adjust and implement, we expect that the reconstruction improvements garnered with localized random sampling have high potential for future application in engineered sampling devices crucial to brain imaging or more general image processing.

In terms of both theoretical implications and directions for future study, the results presented in this paper have several interesting consequences. It would be informative, for example, to derive theoretical error bounds on the reconstruction quality corresponding to different numbers of sampling units and the sparsity structure of the image. On a similar note, extending theoretical arguments regarding the probability of successful reconstruction from simpler independent identically distributed random variable-type sampling to sampling protocols analogous to localized random sampling may signify a useful new direction for CS theory. This may also help to further address the question of to what extent image measurements need to be random for successful CS reconstructions.

With respect to sensory systems, we hypothesize that evolution has selected for sensory sampling schemes analogous to localized random sampling. Given the same number of neurons in the visual system, for example, this work suggests that image information can be encoded more accurately by using sampling protocols alternative to uniformly-random sampling. The center-surround receptive field architecture, prominent in the visual, somatosensory, auditory, and olfactory systems, is akin to localized random sampling in the sense that neurons are most stimulated by a particular range of similar stimuli^12^,^13^,^28^,^29^,^30^,^31^,^32^. In the visual system, this translates to a given ganglion cell sampling spatially clustered image features, with the response depending on where the light falls in the receptive field, i.e., in the center or surround area, as well as the light intensity. Moreover, the size of receptive fields varies widely within a given sensory system and, depending on the receptive field size, details of various scales are measured, just as in the case of varying the *σ* parameter in our localized random sampling protocol^33^,^34^,^35^. Together, the receptive fields of neurons in some layers of the visual system have been shown to form a rough map of visual space, with regions of overlap between the receptive fields varying in size^36^,^37^,^38^. It would be interesting to further extend our localized random sampling by incorporating more specific, detailed structure embedded in the receptive fields of neurons, such as center-surround antagonism and the alignment of receptive fields across different neurons to achieve certain functions, e.g., orientation selectivity.

In many sensory system areas, the number of downstream neurons is significantly less than the number of upstream neurons, such as in the case of downstream ganglion cells and upstream photoreceptors in the retina^39^,^40^. For successful preservation of sensory information across such highly convergent pathways, it is necessary for neuronal network architecture to facilitate efficient signal processing. It is hypothesized that the sparsity of natural scenes combined with the efficiency of localized receptive field sampling may allow for compression of stimulus information along convergent downstream sensory pathways^19^,^20^. This suggests that sampling schemes more precisely resembling these biological architectures may yield further improved information acquisition and retention, which may help to guide future enhancements in efficient image processing. In downstream layers of the visual system, such as the visual cortex, receptive field structure becomes more complex, as input from neurons in upstream layers are integrated, facilitating sensitivity to more complicated characteristics, including feature orientation and direction of motion^33^,^41^,^42^. Thus, multi-layer sampling, yielding more complicated receptive field structures as found in the visual system, may further improve upon CS reconstruction quality.

While we have analyzed the consequences of localized random sampling in particular, similar analysis may be used to understand alternative sampling procedures and their implications on the CS reconstructions. For specific classes of images exhibiting a particular set of features, specialized sampling protocols may also be developed and optimized to yield high quality reconstructions. We expect that localized random sampling in particular may also be useful in reconstructing color images and other classes of sparse signals as well.

## Additional Information

**How to cite this article**: Barranca, V. J. *et al.* Improved Compressive Sensing of Natural Scenes Using Localized Random Sampling. *Sci. Rep.* **6**, 31976; doi: 10.1038/srep31976 (2016).
